# Mortality and cause of death in physical activity and insufficient physical activity participants: a longitudinal follow-up study using a national health screening cohort

**DOI:** 10.1186/s12889-020-09564-x

**Published:** 2020-09-29

**Authors:** Chanyang Min, Dae Myoung Yoo, Jee Hye Wee, Hyo-Jeong Lee, Soo Hwan Byun, Hyo Geun Choi

**Affiliations:** 1 Hallym Data Science Laboratory, Hallym University College of Medicine, Anyang, South Korea; 2 Graduate School of Public Health, Seoul National University, Seoul, South Korea; 3 Department of Otorhinolaryngology-Head & Neck Surgery, Hallym University College of Medicine, Anyang, South Korea; 4 Department of Oral & Maxillofacial Surgery, Dentistry, Sacred Heart Hospital, Hallym University College of Medicine, Anyang, South Korea

**Keywords:** Physical activity, Exercise, Mortality, Obesity, Korea

## Abstract

**Background:**

Few studies have examined the association between physical activity (PA) and various causes of mortality in Korea. The aim of our study was to evaluate mortality and causes of death between PA and insufficient PA using Korean national cohort data.

**Methods:**

The health screening cohort data from the Korean National Health Insurance Service-National Sample Cohort from 2009 to 2015 were used. ‘PA’ was determined if participants walked or performed moderate-intensity activity ≥5 d/week for ≥30 min, or vigorous-intensity activity ≥3 d/week for ≥20 min. Other participants were classified as ‘insufficient PA’. The PA and insufficient PA groups were matched by age, sex, income, and region of residence in a 1:1 ratio. Causes of death were classified into 13 categories. Crude and adjusted hazard ratios (HRs) with 95% confidence intervals (CIs) for all mortality rates were analyzed using a stratified Cox proportional hazard model. Age, sex, income, and region of residence were stratified. Subgroup analyses were performed according to age, sex, and obesity status. The odds ratio according to the causes of death was calculated by the chi-square test.

**Results:**

The adjusted HR for mortality in the PA group was 0.85 (95% CI = 0.82–0.88). In subgroup analyses according to age, sex, and obesity status, results were consistent with the main findings in < 60-year-old females, ≥ 60-year-old males and females, and in all subgroups by obesity. The death rate by neoplasm, mental diseases, neurologic disease, circulatory disease, respiratory disease, digestive disease, abnormal finding, and trauma were lower in the PA than the insufficient PA group.

**Conclusions:**

These results suggest that PA is inversely associated with mortality caused specifically by diseases reflected by mental, respiratory, cancer, and cardiovascular conditions. Additionally, PA is inversely associated with mortality compared to insufficient PA in all obesity status.

## Background

Physical activity (PA) reduces the risk of diseases, such as type 2 diabetes, cardiovascular disease, cancers, musculoskeletal disease, and depression. Moreover, physical inactivity is one of the five leading global risks for mortality [1]. In worldwide, physical inactivity is the main cause or risk factor for approximately 30% of ischemic heart disease burden, 21–25% of breast and colon cancers, and 6% of deaths [2]. Thus, PA can be one of the factors that extends lifespan. Indeed, increasing evidence has demonstrated that PA can decrease mortality, especially mortality from cardiovascular disease and cancer [3, 4]. A review study reported that PA was associated with a risk reduction of 35% (95% confidence intervals [CIs] = 30–40) for mortality due to cardiovascular disease and 33% (95% CI = 28–37) for all-cause mortality [4]. Another review study reported that high levels of PA combined with other positive lifestyle choices resulted in a 50% lower incidence of death from cardiovascular disease, and a similar outcome was found for cancer risk [3].

Likewise, several studies from Korea confirmed that PA could decrease all-cause and certain causes of death. In the Korean Metabolic Syndrome Mortality Study cohort data, exercise could decrease mortality by approximately 17–33% in various cancers, including esophageal, liver, lung, colorectal, and stomach cancers [5]. The all-cause mortality was evaluated according to PAs by adjusting for age, sex, and health-related behavior factors, including smoking, alcohol intake, and nutritional risk, using Korean older adult data [6]. According to the hospital data in Korea, the mortality in Korean men was 37% lower in the regular PA group than in the non-regular PA group [7].

However, only one study has investigated the association between cause specific mortality and PA using cohort data from United State [8]. In addition, no studies have examined the mortality comparing PA and matched insufficient PA groups. The purpose of our study was to identify the mortality and causes of death between PA and insufficient PA groups according to 13 categories based on the International Classification of Diseases, 10th edition (ICD-10) using the health screening cohort from the Korean National Health Insurance Service-National Sample Cohort (NHIS-NSC) data. Mortality was compared between the PA and insufficient PA participants matched at a 1:1 ratio for age, sex, income, and region of residence.

## Methods

### Study population and participant selection

The ethics committee of Hallym University (HALLYM 2019–08-029) permitted the study. Institutional Review Board has waived the written consent.

The data for the health screening cohort from the Korean NHIS-NSC were used. The detail of the Korean NHIS-NSC have been described elsewhere [9, 10]. The data included 514,866 participants who randomly selected 10% from approximately 5,150,000 health insurance holders and who had a health screening by NHIS from 2002 to 2003 in Korea. Those participants were followed up from 2002 to 2015. In 2002 to 2003, the participants were 40 to 79 years old. Among the cohort data, we excluded the participants from 2002 to 2008 because the contents for PA information were different (*n* = 65,180). Hence, we used 449,686 participant data from 2009 to 2015.

**Fig. 1:**
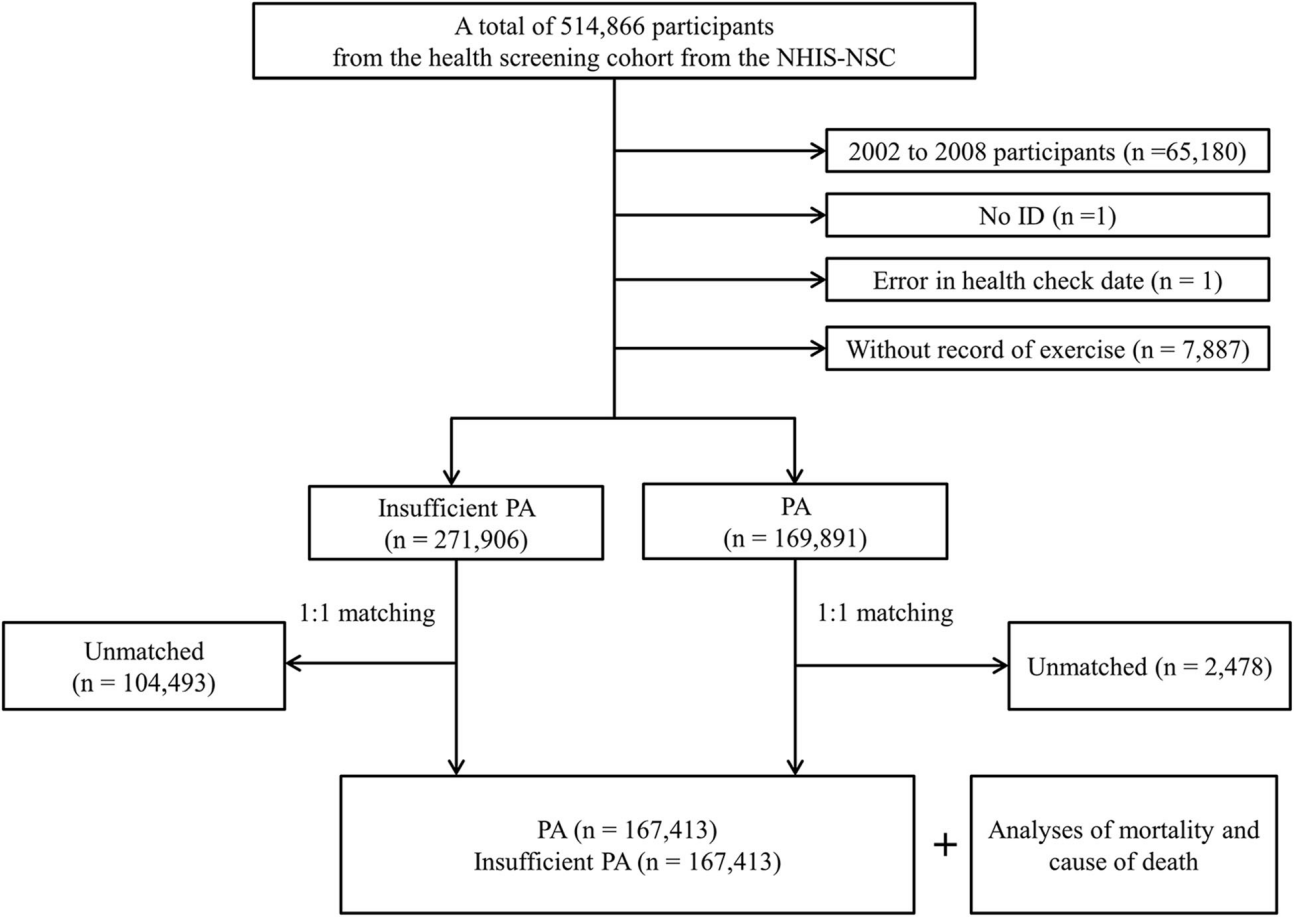
A schematic illustration of the participant selection process that was used in the present study. Of a total of 514,866 participants, 167,413 of physical activity (PA) participants were matched with 167,413 insufficient PA participants for age, sex, income, and region of residence Abbreviations: NHIS-NSC, Korean National Health Insurance Service-National Sample Cohort; PA, physical activity.

Participants were excluded if the ID was missing (*n* = 1), the records for the health checkup date had an error (*n =* 1), or the participants did not have PA information (*n* = 7887). In total, 169,891 participants were included in the PA group, and 271,906 participants were included in the insufficient PA group. We used matching method that was used in our previous studies [11, 12]. PA and insufficient PA groups were matched in the ratio of 1:1 by age, sex, income, and region of residence. Insufficient PA participants were sorted by random number order to minimize selection bias. The index date of each PA participant was defined as the time that the information was collected. Each insufficient PA participants were assigned the same index date as each matched PA participant. During the matching process, 2478 PA participants and 104,493 insufficient PA participants were excluded. Finally, 167,413 participants in the PA group were matched at a 1:1 ratio with 167,413 participants in the insufficient PA group (Fig. 1).

### Determination of physical activity and insufficient physical activity

The PA information was collected using a modified International Physical Activity Questionnaire (IPAQ) [13]. We used PA information from the first record of health screening. ‘PA’ was defined as participants who walked ≥5 d/week for ≥30 min, performed a moderate-intensity activity ≥5 d/week for 30 min, or performed a vigorous-intensity activity ≥3 d/week for 20 min, referring to the IPAQ. Other participants were classified in the ‘insufficient PA’ group.

### Classification of causes of death

The classification of cause specific mortality has been described elsewhere [14]. The casus of death were classified into 12 categories according to the Korean standard classification of diseases which is based on the ICD-10 codes (Additional file 1). Additionally, participants were classified as ‘others’ in our study if their death code was as follows: D50–D89 (diseases of the blood and blood-forming organs and certain disorders involving the immune mechanism); L00–L99 (diseases of the skin and subcutaneous tissue); Q44 or Q61 (other malformations); or missing.

### Covariates

Regarding the age group category, because the data were first collected from 2002 to 2003 from individuals aged 40 to 79 years, the age of participants was ≥45 years old in 2008. Therefore, age group was classified into 5-year intervals starting from 45 years old (e.g., 45–49, 50–54, …, and 85+ years). Income group and region of residence were categorized as our previous study [15].

Tobacco smoking, alcohol consumption, and obesity based on body mass index (BMI, kg/m^2^) [16] were classified in the same way as the previous study [17].

The Charlson Comorbidity Index (CCI) contains 17 diseases to measure burden of comorbidities. The index score was given to each participant according to the severity and number of diseases. Participants were scored 1, 2, 3, or 6 according to the severity of each comorbidity. If comorbidities were ≥ 2 in one participant, then the scores were summed. Therefore, the higher scores of CCI, the more severe and various were the comorbidities. The CCI was measured as a continuous variable (minimum = 0 [no comorbidity], maximum = 15 [7 to 8 comorbidities]) [18, 19].

### Statistical analyses

The general characteristics of study participants were presented using the chi-square test and independent *t* test.

A comparison of the cumulative mortality in the PA and insufficient PA groups was visualized using a Kaplan-Meier survival analysis and the statistical significance was examined using the log-rank test.

A stratified Cox proportional hazard model was used to analyze the hazard ratios (HRs) with 95% confidence intervals (CIs) for mortality in the PA group compared to those in the insufficient PA group. In this analysis, the crude and adjusted models (adjusted for obesity, smoking status, alcohol consumption, and CCI scores) were calculated. The analysis was stratified by age, sex, income, and region of residence.

For the subgroup analyses, we re-grouped participants by age and sex (< 60 years old and ≥ 60 years old; males and females) and analyzed the crude and adjusted models with a stratified Cox model.

Additionally, subgroup analyses according to obesity (underweight, normal weight, overweight, obese I, and obese II) were performed using crude and adjusted models (adjusted for age, sex, income, region of residence, smoking status, alcohol consumption, and CCI scores). In these analyses, we used an unstratified Cox model.

Death rates according to cause specific mortality were calculated using the chi-square test. The false discovery rate was used to adjust for incorrect rejections of the null hypothesis. The odds ratios (ORs) with 95% CIs for all-cause mortality and each cause of mortality were calculated using the chi-square test.

Significance for a two-sided test was determined with a *P*-value < 0.05. For statistical analyses, we used SAS version 9.4 (SAS Institute Inc., Cary, NC, USA).

## Results

The mean follow-up period for the PA group was 65.18 months (standard deviation [SD] = 15.69 months) and for insufficient PA group was 64.92 months (SD = 16.05 months).

**Fig. 2:**
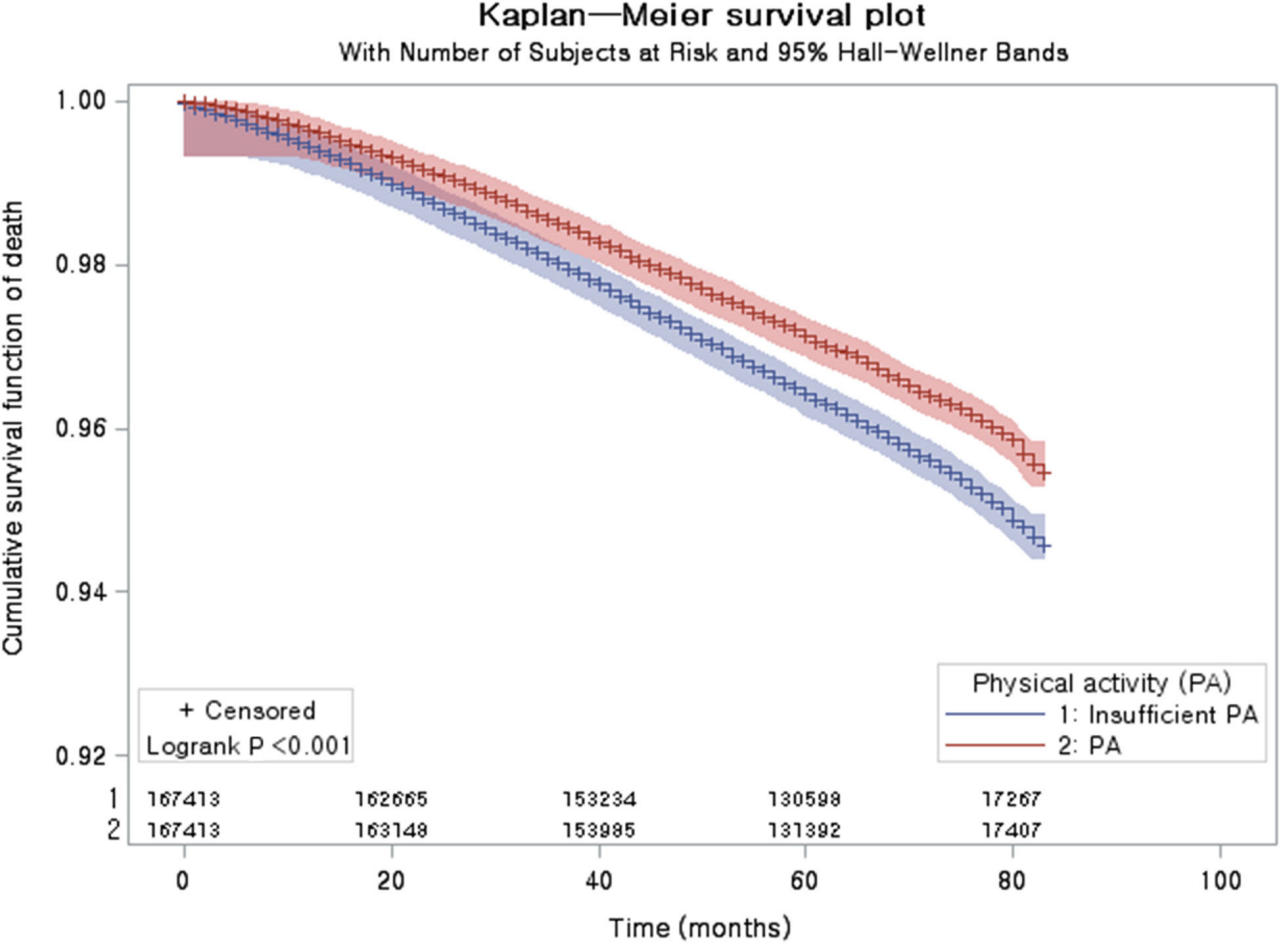
Kaplan-Meier survival analysis and the log-rank test. The cumulative survival rate was lower in the insufficient physical activity (PA) group than in the PA group Abbreviations: PA, physical activity.

The rate of death was significantly lower in the PA group (5483/167,413 [3.3%]) than in the insufficient PA group (6781/167,413 [4.1%], *P* < 0.001, Table 1). The cumulative survival rate was lower (log-rank test, *P* < 0.001) in the insufficient PA group than in the PA group in the Kaplan-Meier survival analysis (Fig. 2).

**Table 1:** General Characteristics of Participants

Characteristics	Total participants		
	PA	Insufficient PA	*P*-value
Age (years old, n, %)			1.000
45–49	17,218 (10.3)	17,218 (10.3)	
50–54	42,312 (25.3)	42,312 (25.3)	
55–59	29,828 (17.8)	29,828 (17.8)	
60–64	31,678 (18.9)	31,678 (18.9)	
65–69	18,687 (11.2)	18,687 (11.2)	
70–74	18,377 (11.0)	18,377 (11.0)	
75–79	6289 (3.8)	6289 (3.8)	
80–84	2664 (1.6)	2664 (1.6)	
85+	360 (0.2)	360 (0.2)	
Sex (n, %)			1.000
Male	93,645 (55.9)	93,645 (55.9)	
Female	73,768 (44.1)	73,768 (44.1)	
Income (n, %)			1.000
1 (lowest)	24,270 (14.5)	24,270 (14.5)	
2	21,864 (13.1)	21,864 (13.1)	
3	25,462 (15.2)	25,462 (15.2)	
4	34,622 (20.7)	34,622 (20.7)	
5 (highest)	61,195 (36.6)	61,195 (36.6)	
Region of residence (n, %)			1.000
Urban	79,044 (47.2)	79,044 (47.2)	
Rural	88,369 (52.8)	88,369 (52.8)	
Obesity (n, %)‡			< 0.001*
Underweight	3331 (2.0)	3778 (2.3)	
Normal	58,361 (34.9)	58,312 (34.8)	
Overweight	48,046 (28.7)	46,304 (27.7)	
Obese I	53,294 (31.8)	54,000 (32.3)	
Obese II	4381 (2.6)	5019 (3.0)	
Smoking status (n, %)			< 0.001*
Nonsmoker	105,936 (63.3)	115,095 (68.8)	
Past smoker	34,938 (20.9)	21,688 (13.0)	
Current smoker	26,539 (15.9)	30,630 (18.3)	
Alcohol consumption			< 0.001*
< 1 time a week	96,476 (57.6)	112,334 (67.1)	
≥ 1 time a week	70,937 (42.4)	55,079 (32.9)	
CCI score (mean, SD)	0.58 (1.29)	0.62 (1.32)	< 0.001†
Death	5483 (3.3)	6781 (4.1)	< 0.001*
Age < 60 years old, males	853 (1.7)	841 (1.7)	0.769
Age < 60 years old, females	232 (0.6)	266 (0.7)	0.126
Age ≥ 60 years old, males	3296 (7.7)	4165 (9.7)	< 0.001*
Age ≥ 60 years old, females	1102 (3.1)	1509 (4.3)	< 0.001*

Abbreviations: *CCI* Charlson comorbidity index, *PA* physical activity

* Chi-square test. Significance at *P* < 0.05

† Independent *t* test. Significance at *P* < 0.05

‡ Obesity (BMI, body mass index, kg/m^2^) was categorized as < 18.5 (underweight), ≥ 18.5 to < 23 (normal), ≥ 23 to < 25 (overweight), ≥ 25 to < 30 (obese I), and ≥ 30 (obese II)

The adjusted HR for mortality in the PA group was significantly lower than the insufficient PA group (0.85, 95% CI = 0.82–0.88, *P <* 0.001). In subgroup analyses among age and sex subgroups, the results were consistent with the above findings in < 60 years old female group and ≥ 60 years old male and female groups (*P <* 0.05, Table 2).

**Table 2:** Crude and adjusted hazard ratios (95% confidence interval) for mortality in the physical activity (PA) group compared with the insufficient PA group with subgroup analyses according to age and sex

Characteristics	Hazard ratios			
	Crude†	*P*-value	Adjusted†‡	*P*-value
Total participants (*n* = 334,826)				
PA	0.79 (0.77–0.82)	< 0.001*	0.85 (0.82–0.88)	< 0.001*
Insufficient PA	1.00		1.00	
Age < 60 years old, males (*n* = 101,708)				
PA	1.01 (0.92–1.12)	0.772	1.05 (0.95–1.16)	0.323
Insufficient PA	1.00		1.00	
Age < 60 years old, females (*n* = 77,008)				
PA	0.87 (0.73–1.04)	0.127	0.81 (0.67–0.96)	0.018*
Insufficient PA	1.00		1.00	
Age ≥ 60 years old, males (*n* = 85,582)				
PA	0.77 (0.74–0.81)	< 0.001*	0.84 (0.80–0.88)	< 0.001*
Insufficient PA	1.00		1.00	
Age ≥ 60 years old, females (*n* = 70,528)				
PA	0.72 (0.66–0.78)	< 0.001*	0.78 (0.72–0.84)	< 0.001*
Insufficient PA	1.00		1.00	

Abbreviations: *CCI* Charlson comorbidity index, *PA* physical activity

* Stratified Cox-proportional hazard regression model, significance at *P* < 0.05

† Models stratified by age, sex, income, and region of residence

‡ A model adjusted for obesity, smoking, alcohol consumption, and CCI scores

Subgroup analyses according to obesity were performed. The adjusted HRs for mortality were lower in the PA group than in the insufficient PA group in all obesity subgroups (*P* < 0.01, Table 3).

**Table 3:** Subgroup analyses of crude and adjusted hazard ratios (95% confidence interval) for mortality in the physical activity (PA) group compared with the insufficient PA group according to obesity

Characteristics	Hazard ratios			
	Crude	*P*-value	Adjusted†	*P*-value
Underweight (*n* = 7109)				
PA	0.77 (0.67–0.88)	< 0.001*	0.83 (0.72–0.95)	0.008*
Insufficient PA	1.00		1.00	
Normal weight (*n* = 116,673)				
PA	0.81 (0.77–0.86)	< 0.001*	0.85 (0.81–0.90)	< 0.001*
Insufficient PA	1.00		1.00	
Overweight (*n* = 94,350)				
PA	0.85 (0.79–0.91)	< 0.001*	0.90 (0.84–0.97)	0.005*
Insufficient PA	1.00		1.00	
Obese I (*n* = 107,294)				
PA	0.80 (0.75–0.86)	< 0.001*	0.80 (0.75–0.86)	< 0.001*
Insufficient PA	1.00		1.00	
Obese II (*n* = 9400)				
PA	0.63 (0.50–0.79)	< 0.001*	0.72 (0.57–0.92)	0.007*
Insufficient PA	1.00		1.00	

Abbreviations: *CCI* Charlson comorbidity index, *PA* physical activity

* Unstratified Cox-proportional hazard regression model, significance at *P* < 0.05

† A model adjusted for age, sex, income, region of residence, smoking, alcohol consumption, and CCI scores

The OR for all-cause mortality was 0.80 (95% CI = 0.77–0.83, *P* < 0.001) in the PA group. Mortality caused by a neoplasm, mental disease, neurologic disease, circulatory disease, respiratory disease, digestive disease, abnormal finding, and trauma was lower in the PA group than the insufficient PA group (*P* < 0.05). The OR for mortality was the lowest for mental disease (0.47, 95% CI = 0.31–0.70, *P* = 0.001) in the PA group. Mortality caused by infection, metabolic disease, muscular disease, and genitourinary disease was not statistically significant between the PA and insufficient PA groups (Table 4). Specific causes of death rates are presented in the Additional file 2.

**Table 4:** The difference in mortality between the physical activity (PA) and insufficient PA groups according to cause of death

Cause of death	Total participants			
	PA(*n* = 167,413)	Insufficient PA (*n* = 167,413)	Odds ratio (95% CI)	*P*-value
All-cause death (n,%)	5483 (100.0)	6781 (100.0)	0.80 (0.77–0.83)	< 0.001*
Infection (n,%)	110 (2.0)	142 (2.1)	0.78 (0.60–0.99)	0.057
Neoplasm (n,%)	2425 (44.2)	2781 (41.0)	0.87 (0.82–0.92)	< 0.001*
Metabolic disease (n,%)	165 (3.0)	202 (3.0)	0.82 (0.67–1.00)	0.063
Mental disease (n,%)	33 (0.6)	71 (1.1)	0.47 (0.31–0.70)	0.001*
Neurologic disease (n,%)	110 (2.0)	155 (2.3)	0.71 (0.56–0.91)	0.010*
Circulatory disease (n,%)	1011 (18.4)	1311 (19.3)	0.77 (0.71–0.84)	< 0.001*
Respiratory disease (n,%)	372 (6.8)	567 (8.4)	0.66 (0.58–0.75)	< 0.001*
Digestive disease (n,%)	171 (3.1)	215 (3.2)	0.80 (0.65–0.97)	0.036*
Muscular disease (n,%)	30 (0.6)	35 (0.5)	0.86 (0.53–1.40)	0.535
Genitourinary disease (n,%)	79 (1.4)	87 (1.3)	0.91 (0.67–1.23)	0.535
Abnormal finding (n,%)	283 (5.2)	365 (5.4)	0.78 (0.66–0.91)	0.002*
Trauma (n,%)	669 (12.2)	801 (11.8)	0.84 (0.75–0.93)	0.002*
Others (n,%)	25 (0.5)	49 (0.7)	0.51 (0.32–0.83)	0.009*

Abbreviation: *PA* physical activity

* Chi-square test. Significance at false discovery rate-adjusted *P* < 0.05

## Discussion

We confirmed the association of mortality by matching PA and insufficient PA participants at a 1:1 ratio by age, sex, income, and region of residence using Korean national cohort data. In addition, we investigated various causes of death, including cancer (neoplasm), circulatory disease, mental disease, and respiratory disease compared between the PA and insufficient PA groups. We found that the all-cause mortality in the PA group was lower than in the insufficient PA group, specifically in female of all age groups, in older male group, and in all obesity groups. Among the causes of death, mental disease had the lowest mortality in the PA group compared with the insufficient PA group. In addition, respiratory disease, neurologic disease, circulatory disease, digestive disease, abnormal finding, trauma, and neoplasm (cancer) mortalities were also lower in the PA group than the insufficient PA group.

In our study, due to the lack of information from secondary data, PA information was not specifically collected according to the length of time of each intensity of PA. Although we grouped participants into the PA group based on the broad definition refer to the IPAQ criteria [13], the PA group was associated with a lower risk of mortality than with the insufficient PA group. A review study has suggested that a large amount of PA exceeding the recommended amount of PA is better to lower the mortality risk [20]. Conversely, one study demonstrated that light-intensity PA (3 to < 9 metabolic equivalents [METs] hours/week) in older adults (aged 50 to 74 years) may be associated with a lower risk of mortality than with little/no light-intensity PA. Moreover, the study reported that all-cause mortality was not significantly different between light PA and other higher intensity PAs [21]. Therefore, the findings from the above studies can explain the simplification of the PA group in our study. We additionally performed subgroup analyses according to the types of PA performed by the participants (walking, moderate-intensity activity, vigorous-intensity activity, and other combinations of PAs) with each matched insufficient PA participant. As suggested in previous studies, the risk of all-cause mortality was lower even in the participants who performed walking, a simpler and lighter PA than other types of PA (Additional file 3).

In our study, all-cause mortality including mortality due to circulatory disease (cardiovascular disease) and neoplasm (cancer), was lower in PA group compared with the insufficient PA group. Previous studies reported similar results to our study findings [3–5]. Although the mechanisms between PA and mortality are not fully understood, various benefits of PA that are important predictors of chronic disease such as cardiovascular disease and cancer have been suggested as a link between PA and decreasing mortality. Some related benefits of PA are as follows: changing body composition, anti-inflammatory effects, decreasing biological and psychosocial stressors that optimize neurogenesis, and an improved immune system [22–24].

Mortality due to mental disease, including Alzheimer’s disease and dementia, was the lowest among the causes of mortality in the PA group than the insufficient PA group in our study. The results from previous studies are consistent with our study findings. One study found that mortality was lower in the higher intensity PA groups (≥ 150 min/week for each non-recreational and recreational PA) than the lower intensity PA group (< 150 min/week for each of non-recreational and recreational PA) in Korean Alzheimer’s disease patients (HR = 0.22, 95% CI = 0.05–0.88, *P* = 0.033) [25]. Another study has also reported that improving mental health increases PA [26]. Brain blood flow increases via PA could lead to changes in brain metabolism and increases in neural activation, thus decreasing the onset of dementia [27]. Hence, PA could prevent or improve mental disease and thus may decrease mental disease mortality.

The mortality associated with respiratory diseases, specifically influenza, pneumonia, and chronic lower respiratory diseases, was also lower in the PA group than the insufficient PA group in our study. One study reported that walking ≥2 h/week was associated with a lower respiratory disease mortality [28]. Another study demonstrated that higher amounts of walking or running were associated with a lower risk of respiratory disease mortality [29]. Previous studies suggest that PA could enhance the immune system by increasing anti-influenza IgG and IgM levels or could inhibit lung inflammation by reducing inflammatory cytokines and oxidative stress markers [30, 31]. Based on previous studies and our study result, PA, specifically aerobic PA such as walking, could lower the risk of respiratory disease mortality by enhancing the immune system.

Since most of retirement ages in Korea are 60 years old, we classified the age group as < 60 years and ≥ 60 years old in our study. The risk of mortality was not significant between the PA and insufficient PA groups in males < 60 years old. Conversely, the risk of mortality was significantly lower in the PA group than the insufficient PA group in females < 60 years old. The reason for the inconsistent findings according to sex may be because our study data did not specifically classify occupational PA and leisure time PA in the PA questionnaire. A previous study defined high occupational PA as individuals who were taking stairs > 20 floors or walking > 3 km/day at work, often taking stairs and carrying light stuffs, or exerting a lot of physical effort and frequently carrying heavy stuffs. The study reported that, unlike leisure-time PA, the risk of all-cause mortality and cardiovascular disease mortality was higher in males with a high occupational PA, while no difference was shown among the intensity of occupational PAs in females [32]. The cause of different findings between males and females would be longer and higher intensity work in males than females [33, 34]. Moreover, exceeding the recommended times and intensity of PA, such as over 8 h of high-intensity work, may impair cardiovascular health [35]. Therefore, this so-called PA health paradox [36, 37] may affect the different study findings between males and females.

In all obesity categories, including underweight, the HRs for mortality were lower in the PA group than the insufficient PA group in our study. The evidence demonstrated that BMI has a U-shaped or J-shaped association with higher mortality not only in overweight or obesity, but also in underweight [38]. Although we could not find a study that investigated the association between underweight individuals with physical activity and mortality, the findings from a previous study show that the log hazard of death was higher in sedentary men with lower BMI percentiles than in active men with lower BMI percentiles [39]. Therefore, underweight individuals could improve their health by improving physical activity. Further studies should elucidate the association between PA and mortality in underweight individuals.

The main strength of our study was using large cohort data over a 7-year follow-up period. Moreover, we matched participants in the PA and insufficient PA groups at a 1:1 ratio by age, sex, income, and region of residence to independently compare the causes of mortality. In addition, we conducted subgroup analyses according to obesity status to identify whether PA with all obesity status are negatively associated with mortality. Furthermore, we investigated various causes of mortality between the PA and insufficient PA groups.

Several limitations of our study should be considered. First, due to the use of secondary data, we could not calculate the METs in our study. Therefore, we could not classify the PA group into specific categories. Moreover, we could not follow-up whether the PA status was maintained until the end of the follow-up period because it was not mandatory for the participants to have a health screening every year. In addition, PA was not specifically classified according to whether it was performed during leisure or occupational time. Due to the use of secondary data, only participants aged ≥45 years were included. Regarding BMI, obesity status may not be correct because BMI records were replaced with the mean BMI from the total selected participants for 115 participants with missing BMI records [40]. Moreover, to preserve the number of participants, we were unable to perform a subgroup analysis based on obesity status using the stratified model because the participants in the PA and insufficient PA groups did not match according to their obesity status. Because we performed an observational study, definite causality between PA and mortality could not be determined.

## Conclusion

The results suggest that PA is negatively associated with mortality, including mortality from not only cancer or cardiovascular disease, but also diseases such as mental disease or respiratory disease. In addition, PA is negatively associated with mortality compared with insufficient PA for all of obesity status.

## Supplementary information

**Additional file 1:.** Supplementary file 1. Korean standard classification of diseases based on the ICD-10 codes

**Additional file 2:.** S1Table. Cause of death in the physical activity (PA) and insufficient PA groups

**Additional file 3:.** S2 Table. Subgroup analyses of crude and adjusted hazard ratios (95% confidence interval) for mortality in the physical activity (PA) group compared with the insufficient PA group according to types of exercise

## Data Availability

The data that support the findings of this study are available from the NHISS and all researchers can apply for using the database to conduct their studies. Permission from the NHIS is required to access the database. However, restrictions apply to the availability of these data due to ethical concerns, which were used under license for the current study and so are not publicly available. Any raw data are not allowed to be brought out due to the ethical concerns.

## Abbreviations

CCI: Charlson Comorbidity Index
CIs: Confidence intervals
HRs: Hazard ratios
HURF: Hallym University Research Fund
ICD-10: International Classification of Diseases, 10th edition
IPAQ: International Physical Activity Questionnaire
METs: Metabolic equivalents
NHIS: National Health Insurance Service
NHIS-NSC: National Health Insurance Service-National Sample Cohort
NHISS: Korean National Health Insurance Sharing Service
NRF: National Research Foundation
ORs: Odds ratios
PA: Physical activity
SD: Standard deviation
