# Supplementary material for: Mortality and cause of death in physical activity and insufficient physical activity participants: a longitudinal follow-up study using a national health screening cohort
Source: BMC Public Health. 2020 Sep 29;20:1469. doi: 10.1186/s12889-020-09564-x (PMC7526194; doi:10.1186/s12889-020-09564-x)
Supplement: Supplementary file 1 — Additional file 1:. Supplementary file 1. Korean standard classification of diseases based on the ICD-10 codes [file 12889_2020_9564_MOESM1_ESM.docx]

**Korean standard classification of diseases based on the ICD-10 codes**

(1) infection (certain infections and parasitic diseases, A00–B99)

(2) neoplasm (neoplasms, C00–D48)

(3) metabolic disease (endocrine, nutritional and metabolic diseases, E00–E90)

(4) mental disease (mental and behavioral disorders, F00–F99)

(5) neurologic disease (diseases of the nervous system, G00–G99)

(6) circulatory disease (diseases of the circulatory system, I00–I99)

(7) respiratory disease (diseases of the respiratory system, J00–J99)

(8) digestive disease (diseases of the digestive system, K00–K93)

(9) muscular disease (diseases of the musculoskeletal system and connective tissue, M00–M99)

(10) genitourinary disease (diseases of the genitourinary system, N00–N99)

(11) abnormal finding (symptoms, signs and abnormal clinical and laboratory findings ‘not elsewhere classified’, R00–R99)

(12) trauma (injury, poisoning, and certain other consequences of external causes, S00–T98).
