# Supplementary material for: Mortality and cause of death in physical activity and insufficient physical activity participants: a longitudinal follow-up study using a national health screening cohort
Source: BMC Public Health. 2020 Sep 29;20:1469. doi: 10.1186/s12889-020-09564-x (PMC7526194; doi:10.1186/s12889-020-09564-x)
Supplement: Supplementary file 2 — Additional file 2:. S1Table. Cause of death in the physical activity (PA) and insufficient PA groups [file 12889_2020_9564_MOESM2_ESM.docx]

**S1Table** Cause of death in the physical activity (PA) and insufficient PA groups

| Cause of death | | Codes | No. of Death | |
| --- | --- | --- | --- | --- |
|  |  |  | PA  (n = 5,483) | Insufficient PA (n = 6,781) |
| **Infection** | | **A00-B99** | **110** | **142** |
|  | Intestinal Infectious Diseases | A00-A09 | 5 | 7 |
|  | Tuberculosis | A15-A19 | 45 | 38 |
|  | Certain Zoonotic Bacterial diseases | A20-A28 | 0 | 0 |
|  | Other bacterial diseases | A30-A49 | 24 | 51 |
|  | Infections with a predominantly sexual mode of transmission | A50-A64 | 0 | 0 |
|  | Other spirochetal diseases | A65-A69 | 0 | 0 |
|  | Other diseases caused by chlamydia | A70-A74 | 0 | 0 |
|  | Rickettsioses | A75-A79 | 2 | 3 |
|  | Viral infections of the central nervous system | A80-A89 | 4 | 1 |
|  | Arthropod-borne viral fevers and viral hemorrhagic fevers | A92-A99 | 0 | 1 |
|  | Viral infections characterized by skin and mucous membrane lesions | B00-B09 | 1 | 1 |
|  | Viral hepatitis | B15-B19 | 20 | 25 |
|  | Human immunodeficiency virus[HIV] disease | B20-B24 | 2 | 5 |
|  | Other viral diseases | B25-B34 | 0 | 2 |
|  | Mycoses | B35-B49 | 4 | 1 |
|  | Protozoal diseases | B50-B64 | 0 | 0 |
|  | Helminthiases | B65-B83 | 0 | 0 |
|  | Pediculosis, acariasis and other infestations | B85-B89 | 0 | 0 |
|  | Sequelae of infectious and parasitic diseases | B90-B94 | 3 | 6 |
|  | Bacterial, viral and other infectious agents | B95-B98 | 0 | 0 |
|  | Other infectious diseases | B99 | 0 | 1 |
| **Neoplasm** | | **C00-D48** | **2,425** | **2,781** |
|  | Malignant neoplasm of lip, oral cavity and pharynx | C00-C14 | 29 | 30 |
|  | Malignant neoplasms of digestive organs | C15-C26 | 1,258 | 1,399 |
|  | Malignant neoplasms of respiratory and intrathoracic organs | C30-C39 | 603 | 830 |
|  | Malignant neoplasm of bone and articular cartilage | C40-C41 | 1 | 3 |
|  | Melanoma and other malignant neoplasms of skin | C43-C44 | 14 | 8 |
|  | Malignant neoplasms of mesothelial and soft tissue | C45-C49 | 15 | 17 |
|  | Malignant neoplasm of breast | C50 | 29 | 34 |
|  | Malignant neoplasm of female genital organs | C51-C58 | 45 | 45 |
|  | Malignant neoplasm of male genital organs | C60-C63 | 67 | 74 |
|  | Malignant neoplasm of urinary tract | C64-C68 | 96 | 74 |
|  | Malignant neoplasm of eye, brain and other parts of central nervous system | C69-C72 | 30 | 32 |
|  | Malignant neoplasm of thyroid and other endocrine gland | C73-C75 | 16 | 12 |
|  | Malignant neoplasm of ill-defined, secondary and unspecified sites | C76-C80 | 28 | 34 |
|  | Malignant neoplasms of lymphoid, hematopoietic and related tissue | C81-C96 | 168 | 153 |
|  | Malignant neoplasm of independent (primary) multiple sites | C97 | 1 | 3 |
|  | In situ neoplasms | D00-D09 | 0 | 0 |
|  | Benign neoplasms | D10-D36 | 3 | 3 |
|  | Neoplasms of uncertain or unknown behavior | D37-D48 | 22 | 30 |
| **Metabolic disease** | | **E00-E90** | **165** | **202** |
|  | Disorders of thyroid gland | E00-E07 | 2 | 0 |
|  | Diabetes mellitus | E10-E14 | 153 | 190 |
|  | Other disorders of glucose regulation and pancreatic internal secretion | E15-E16 | 0 | 1 |
|  | Disorders of other endocrine glands | E20-E35 | 2 | 0 |
|  | Malnutrition | E40-E46 | 1 | 1 |
|  | Other nutritional deficiencies | E50-E64 | 0 | 0 |
|  | Obesity and other hyperalimentation | E65-E68 | 0 | 0 |
|  | Metabolic disorders | E70-E90 | 7 | 10 |
| **Mental disease** | | **F00-F99** | **33** | **71** |
|  | Organic, including symptomatic mental disorders | F00-F09 | 28 | 58 |
|  | Mental and behavioral disorders due to psychoactive substance use | F10-F19 | 5 | 10 |
|  | Schizophrenia, schizotypal and delusional disorders | F20-F29 | 0 | 2 |
|  | Mood [affective] disorders | F30-F39 | 0 | 1 |
|  | Neurotic, stress-related and somatoform disorders | F40-F48 | 0 | 0 |
|  | Behavioral syndromes associated with physiological disturbances and physical factors | F50-F59 | 0 | 0 |
|  | Disorders of adult personality and behavior | F60-F69 | 0 | 0 |
|  | Mental retardation | F70-F79 | 0 | 0 |
|  | Disorders of psychological development | F80-F89 | 0 | 0 |
|  | Behavioral and emotional disorders with onset usually occurring in childhood and adolescents | F90-F98 | 0 | 0 |
|  | Unspecified mental disorder | F99 | 0 | 0 |
| **Neurologic disease** | | **G00-G99** | **110** | **155** |
|  | Inflammatory diseases of the central nervous system | G00-G09 | 6 | 7 |
|  | Systemic atrophies primarily affecting the central nervous system | G10-G14 | 9 | 15 |
|  | Extrapyramidal and movement disorders | G20-G26 | 59 | 73 |
|  | Other degenerative diseases of the nervous system | G30-G32 | 26 | 37 |
|  | Demyelinating diseases of the central nervous system | G35-G37 | 1 | 1 |
|  | Episodic and paroxysmal disorders | G40-G47 | 2 | 8 |
|  | Nerve, nerve root and plexus disorders | G50-G59 | 0 | 0 |
|  | Polyneuropathies and other disorders of the peripheral nervous system | G60-G64 | 0 | 0 |
|  | Diseases of myoneural junction and muscle | G70-G73 | 1 | 0 |
|  | Cerebral palsy and other paralytic syndromes | G80-G83 | 0 | 1 |
|  | Other disorders of the nervous system | G90-G99 | 6 | 13 |
| **Circulatory disease** | | **I00-I99** | **1,011** | **1,311** |
|  | Acute rheumatic fever | I00-I02 | 0 | 0 |
|  | Chronic rheumatic heart diseases | I05-I09 | 1 | 4 |
|  | Hypertensive diseases | I10-I15 | 48 | 71 |
|  | Ischemic heart diseases | I20-I25 | 316 | 373 |
|  | Pulmonary heart disease and diseases of pulmonary circulation | I26-I28 | 7 | 8 |
|  | Other forms of heart disease | I30-I52 | 191 | 243 |
|  | Cerebrovascular diseases | I60-I69 | 415 | 563 |
|  | Diseases of arteries, arterioles and capillaries | I70-I79 | 29 | 42 |
|  | Diseases of veins, lymphatic vessels and lymph nodes, NEC | I80-I89 | 4 | 5 |
|  | Other and unspecified disorders of the circulatory system | I95-I99 | 0 | 2 |
| **Respiratory disease** | | **J00-J99** | **372** | **567** |
|  | Acute upper respiratory infections | J00-J06 | 1 | 0 |
|  | Influenza and pneumonia | J09-J18 | 148 | 229 |
|  | Other acute lower respiratory infections | J20-J22 | 0 | 2 |
|  | Other diseases of upper respiratory tract | J30-J39 | 1 | 0 |
|  | Chronic lower respiratory diseases | J40-J47 | 125 | 200 |
|  | Lung diseases due to external agents | J60-J70 | 33 | 52 |
|  | Other respiratory diseases principally affecting the interstitium | J80-J84 | 45 | 60 |
|  | Suppurative and necrotic conditions of lower respiratory tract | J85-J86 | 2 | 6 |
|  | Other diseases of pleura | J90-J94 | 5 | 1 |
|  | Other diseases of the respiratory system | J95-J99 | 12 | 17 |
| **Digestive disease** | | **K00-K93** | **171** | **215** |
|  | Diseases of oral cavity, salivary glands and jaws | K00-K14 | 0 | 0 |
|  | Diseases of esophagus, stomach and duodenum | K20-K31 | 8 | 16 |
|  | Disease of appendix | K35-K38 | 2 | 1 |
|  | Hernia | K40-K46 | 0 | 1 |
|  | Noninfective enteritis and colitis | K50-K52 | 1 | 3 |
|  | Other diseases of intestines | K55-K64 | 14 | 28 |
|  | Diseases of peritoneum | K65-K67 | 3 | 5 |
|  | Diseases of liver | K70-K77 | 121 | 125 |
|  | Disorders of gallbladder, biliary tract and pancreas | K80-K87 | 16 | 28 |
|  | Other diseases of the digestive system | K90-K93 | 6 | 8 |
| **Muscular disease** | | **M00-M99** | **30** | **35** |
|  | Infectious arthropathies | M00-M03 | 2 | 0 |
|  | Inflammatory polyarthropathies | M05-M14 | 3 | 7 |
|  | Arthrosis | M15-M19 | 1 | 0 |
|  | Other joint disorders | M20-M25 | 0 | 0 |
|  | Systemic connective tissue disorder | M30-M36 | 6 | 6 |
|  | Deforming dorsopathies | M40-M43 | 0 | 0 |
|  | Spondylopathies | M45-M49 | 5 | 5 |
|  | Other dorsopathies | M50-M54 | 0 | 1 |
|  | Disorders of muscles | M60-M63 | 3 | 3 |
|  | Disorders of synovium and tendon | M65-M68 | 0 | 0 |
|  | Other soft tissue disorders | M70-M79 | 2 | 3 |
|  | Disorders of bone density and structure | M80-M85 | 7 | 9 |
|  | Other osteopathies | M86-M90 | 1 | 1 |
|  | Chondropathies | M91-M94 | 0 | 0 |
|  | Other disorders of the musculoskeletal system and connective tissue | M95-M99 | 0 | 0 |
| **Genitourinary disease** | | **N00-N99** | **79** | **87** |
|  | Glomerular diseases | N00-N08 | 2 | 0 |
|  | Renal tubulo-interstitial diseases | N10-N16 | 4 | 4 |
|  | Renal failure | N17-N19 | 66 | 77 |
|  | Urolithiasis | N20-N23 | 0 | 1 |
|  | Other disorders of kidney and ureter | N25-N29 | 0 | 0 |
|  | Other diseases of the urinary system | N30-N39 | 7 | 5 |
|  | Diseases of male genital organs | N40-N51 | 0 | 0 |
|  | Disorders of breast | N60-N64 | 0 | 0 |
|  | Inflammatory diseases of female pelvic organs | N70-N77 | 0 | 0 |
|  | Noninflammatory disorders of female genital tract | N80-N98 | 0 | 0 |
|  | Other disorders of the genitourinary system | N99 | 0 | 0 |
| **Abnormal finding** | | **R00-R99** | **283** | **365** |
|  | Symptoms and signs involving the circulatory and respiratory systems | R00-R09 | 17 | 29 |
|  | Symptoms and signs involving the digestive system and abdomen | R10-R19 | 0 | 0 |
|  | Symptoms and signs involving the skin and subcutaneous tissue | R20-R23 | 0 | 0 |
|  | symptoms and signs involving the nervous and musculoskeletal systems | R25-R29 | 0 | 0 |
|  | symptoms and signs involving the urinary system | R30-R39 | 0 | 0 |
|  | Symptoms and signs involving cognition, perception, emotional state and behavior | R40-R46 | 0 | 1 |
|  | Symptoms and signs involving speech and voice | R47-R49 | 0 | 0 |
|  | General symptoms and signs | R50-R69 | 129 | 185 |
|  | Abnormal findings on examination of blood, without diagnosis | R70-R79 | 0 | 0 |
|  | Abnormal findings on examination of urine, without diagnosis | R80-R82 | 0 | 0 |
|  | Abnormal findings on examination of other body fluids, substances and tissues, without diagnosis | R83-R89 | 0 | 0 |
|  | Abnormal findings on diagnostic imaging and in function studies, without diagnosis | R90-R94 | 0 | 0 |
|  | Ill-defined and unknown causes of mortality | R95-R99 | 137 | 150 |
| **Trauma** | | **S00-T98** | **669** | **801** |
|  | Injuries to the head | S00-S09 | 153 | 160 |
|  | Injuries to the neck | S10-S19 | 5 | 12 |
|  | Injuries to the thorax | S20-S29 | 27 | 39 |
|  | Injuries to the abdomen, lower back, lumbar spine and pelvis | S30-S39 | 15 | 20 |
|  | Injuries to the shoulder and upper arm | S40-S49 | 0 | 0 |
|  | Injuries to the elbow and forearm | S50-S59 | 0 | 0 |
|  | Injuries to the wrist and hand | S60-S69 | 1 | 2 |
|  | Injuries to the hip and thigh | S70-S79 | 15 | 16 |
|  | Injuries to the knee and lower leg | S80-S89 | 2 | 1 |
|  | Injuries to the ankle and foot | S90-S99 | 0 | 0 |
|  | Injuries involving multiple body regions | T00-T07 | 113 | 116 |
|  | Injuries to unspecified part of trunk, limb or body region | T08-T14 | 31 | 39 |
|  | Effects of foreign body entering through natural orifice | T15-T19 | 10 | 8 |
|  | Burns and corrosions of external body surface, specified by site | T20-T25 | 1 | 0 |
|  | Burn and corrosions confined to eye and internal organs | T26-T28 | 0 | 0 |
|  | Burns and corrosions of multiple and unspecified of multiple and unspecified body regions | T29-T32 | 4 | 6 |
|  | Frostbite | T33-T35 | 0 | 0 |
|  | Poisoning by drugs, medicaments and biological substances | T36-T50 | 3 | 3 |
|  | Toxic effects of substances chiefly nonmedicinal as to source | T51-T65 | 86 | 135 |
|  | Other and unspecified effects of external causes | T66-T78 | 189 | 225 |
|  | Certain early complications of trauma | T79 | 3 | 6 |
|  | Complications of surgical and medical care, NEC | T80-T88 | 2 | 7 |
|  | Sequelae of injures, of poisoning and of other consequences of external causes | T90-T98 | 9 | 6 |
| **Others** | |  | **25** | **49** |
|  | Nutritional anemias | D50-D53 | 1 | 1 |
|  | Hemolytic anemias | D55-D59 | 1 | 1 |
|  | Aplastic and other anemias | D60-D64 | 2 | 6 |
|  | Coagulation defects, purpura and other hemorrhage conditions | D65-D69 | 1 | 3 |
|  | Other disease of blood and blood-forming organs | D70-D77 | 3 | 5 |
|  | Diseases of the skin and subcutaneous tissue | L00-L99 | 3 | 3 |
|  | Other malformations | Q44, Q61 | 0 | 4 |
|  | Missing |  | 15 | 26 |

Abbreviations: PA, physical activity
