# Supplementary material for: Mortality and cause of death in physical activity and insufficient physical activity participants: a longitudinal follow-up study using a national health screening cohort
Source: BMC Public Health. 2020 Sep 29;20:1469. doi: 10.1186/s12889-020-09564-x (PMC7526194; doi:10.1186/s12889-020-09564-x)
Supplement: Supplementary file 3 — Additional file 3:. S2 Table. Subgroup analyses of crude and adjusted hazard ratios (95% confidence interval) for mortality in the physical activity (PA) group compared with the insufficient PA group according to types of exercise [file 12889_2020_9564_MOESM3_ESM.docx]

**S2 Table** Subgroup analyses of crude and adjusted hazard ratios (95% confidence interval) for mortality in the physical activity (PA) group compared with the insufficient PA group according to types of exercise

| Characteristics | | Hazard ratios | | | |
| --- | --- | --- | --- | --- | --- |
|  |  | Crude† | P-value | Adjusted‡ | P-value |
| Walking (n = 71,683 for exercise group, n = 71,683 for non-exercise group) | | | | | |
|  | PA | 0.85 (0.81-0.90) | <0.001* | 0.87 (0.83-0.92) | <0.001* |
|  | Insufficient PA | 1.00 |  | 1.00 |  |
| Moderate activity (n = 5,726 for exercise group, n = 5,726 for non-exercise group) | | | | | |
|  | PA | 0.74 (0.61-0.89) | 0.001 | 0.88 (0.72-1.08) | 0.219 |
|  | Insufficient PA | 1.00 |  | 1.00 |  |
| Vigorous activity (n = 40,572 for exercise group, n = 40,572 for non-exercise group) | | | | | |
|  | PA | 0.75 (0.69-0.82) | <0.001* | 0.90 (0.82-0.98) | 0.012* |
|  | Insufficient PA | 1.00 |  | 1.00 |  |
| Walking + moderate activity (n = 10,526 for exercise group, n = 10,526 for non-exercise group) | | | | | |
|  | PA | 0.74 (0.64-0.85) | <0.001* | 0.76 (0.65-0.88) | <0.001* |
|  | Insufficient PA | 1.00 |  | 1.00 |  |
| Walking + vigorous activity (n = 13,905 for exercise group, n = 13,905 for non-exercise group) | | | | | |
|  | PA | 0.72 (0.63-0.82) | <0.001* | 0.82 (0.70-0.94) | 0.006* |
|  | Insufficient PA | 1.00 |  | 1.00 |  |
| Moderate + vigorous activity (n =2,949 for exercise group, n = 2,949 for non-exercise group) | | | | | |
|  | PA | 0.79 (0.58-1.08) | 0.146 | 1.05 (0.74-1.50) | 0.790 |
|  | Insufficient PA | 1.00 |  | 1.00 |  |
| All activities (n = 22,052 for exercise group, n = 22,052 for non-exercise group) | | | | | |
|  | PA | 0.73 (0.66-0.80) | <0.001* | 0.79 (0.72-0.87) | <0.001* |
|  | Insufficient PA | 1.00 |  | 1.00 |  |

Abbreviations: CCI, Charlson comorbidity index; PA, physical activity

* Stratified Cox-proportional hazard regression model, significance at P < 0.05

† Models stratified by age, sex, income, and region of residence.

‡ A model adjusted for obesity, smoking, alcohol consumption, and CCI scores
